# The *Drosophila* RASSF Homolog Antagonizes the Hippo Pathway

**DOI:** 10.1016/j.cub.2006.10.060

**Published:** 2006-12-19

**Authors:** Cedric Polesello, Sven Huelsmann, Nicholas H. Brown, Nicolas Tapon

**Affiliations:** 1 Apoptosis and Proliferation Control Laboratory, Cancer Research UK, London Institute, 44 Lincoln's Inn Fields, London, United Kingdom; 2 The Gurdon Institute and Department of Physiology, Development, and Neuroscience, University of Cambridge, Tennis Court Road, Cambridge, United Kingdom

**Keywords:** SIGNALLING, DEVBIO, CELLCYCLE

## Abstract

Correct organ size is determined by the balance between cell death and proliferation. Perturbation of this delicate balance leads to cancer formation [1]. Hippo (Hpo), the *Drosophila* ortholog of MST1 and MST2 (Mammalian Sterile 20-like 1 and 2) is a key regulator of a signaling pathway that controls both cell death and proliferation 2, 3. This pathway is so far composed of two Band 4.1 proteins, Expanded (Ex) and Merlin (Mer), two serine/threonine kinases, Hpo and Warts (Wts), the scaffold proteins Salvador (Sav) and Mats, and the transcriptional coactivator Yorkie (Yki). It has been proposed that Ex and Mer act upstream of Hpo, which in turn phosphorylates and activates Wts. Wts phosphorylates Yki and thus inhibits its activity and reduces expression of Yki target genes such as the caspase inhibitor DIAP1 and the micro RNA *bantam*^4^, ^5^, ^6^. However, the mechanisms leading to Hpo activation are still poorly understood. In mammalian cells, members of the Ras association family (RASSF) of tumor suppressors have been shown to bind to MST1 and modulate its activity [7]. In this study, we show that the *Drosophila* RASSF ortholog (dRASSF) restricts Hpo activity by competing with Sav for binding to Hpo. In addition, we observe that dRASSF also possesses a tumor-suppressor function.

## Results and Discussion

The mammalian *RASSF* family comprises six different loci encoding a variety of splice variants. Most transcripts encode proteins that contain a Ras association domain (RA), an N-terminal C1-type zinc finger, and a C-terminal SARAH (Sav RASSF Hippo) domain (8, 9, 10, 11, 12, 13 and Figure S1A). RASSF family members, most notably *RASSF1A*, are frequently silenced in a variety of solid tumors [14], mainly by promoter methylation [15]. Thus, it has been proposed that *RASSF* genes act as tumor suppressors.

The biological function of these genes is not well understood. RASSF1A and Nore1A have both been shown to interact with MST1 via its SARAH domain [7]. Overexpression of RASSF1A or Nore1A inhibits MST1 activation, but coexpression of these RASSF proteins with Ras enhanced MST1 activity [16]. *RASSF1A* knockout mice have mildly increased tumor susceptibility [17], confirming that *RASSF* genes can act as tumor suppressors. The weakness of the mouse phenotype, which is at odds with the frequency of RASSF1A inactivation in human tumors, can be ascribed to redundancy with other family members.

By contrast, *Drosophila melanogaster* has a single RASSF family member, which is encoded by the *CG4656* gene and which we will refer to as *dRASSF*. Like its vertebrate counterparts, *dRASSF* encodes a protein bearing an RA and SARAH domain at its C terminus (Figure S1A in the Supplemental Data available online). It also possesses a LIM domain that shares some similarities with C1 zinc fingers at its N terminus.

We generated mutant alleles of *dRASSF* by imprecise excision of two nearby transposons, GE23517 and EY2800 (see Supplemental Experimental Procedures). We obtained multiple alleles, which delete up to the fourth intron, including the initiating ATG (Figure S1B). Some transcript was still detected in *dRASSF^X16^, dRASSF^X36^,* but a strong reduction was found in dRASSF^44.2^, which lacks the transcription start (Figure S1C). However, antibodies raised against the C terminus (amino acids 792–806) and a nonconserved region (amino acids 294–308) of dRASSF showed that full-length dRASSF is absent in lysates from all mutant lines, suggesting our *dRASSF* mutants are indeed loss-of-function mutations for the locus (Figure S1D and data not shown). All of these alleles were viable and behaved identically in subsequent assays. In addition, dRASSF staining was severely reduced in FLP/FRT-generated *dRASSF* mutant clones in the eye-imaginal disc, the larval precursor to the adult eye (Figure S1E).

**Figure 1:**
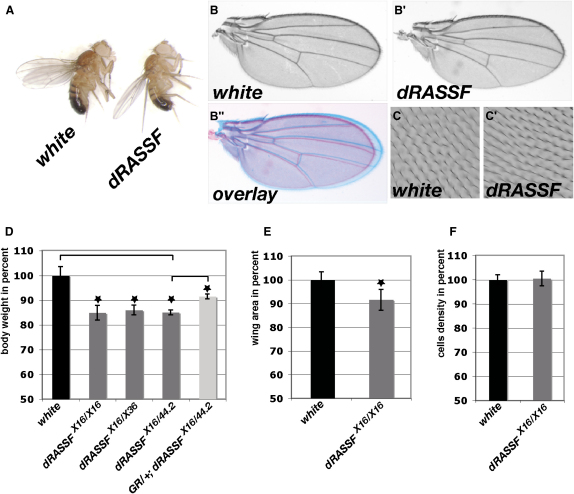
dRASSF Controls Body Size (A) *white* and *dRASSF^X16/X16^* adult flies showing that *dRASSF* flies present a size defect. (B–B″) *dRASSF^X16/X16^* wings (B′) are smaller than *white* wings (B). (B″) Overlay of B and B′. (C and C′) Cell density is not affected in *dRASSF* wings. Phase-contrast image of wing hairs on the wing surface of *white* and *dRASSF^X16/X16^* flies. Note identical hair densities, indicating normal cell size in mutant wings. (D) Histogram representing fly weights as percent of *white* control animals. *dRASSF* flies are 15% smaller than *white* flies. This weight defect is partially rescued in the presence of the genomic rescue construct (GR). ^∗^p < 0.05 (*white* n = 120, *dRASSF^X16/X16^* n = 120, *dRASSF^X16/X36^* n = 90, *dRASSF^X16/44.2^* n = 90, *GR;dRASSF^X16/44.2^* n = 90). (E) Histogram representing wing areas as percent of control (*white*) wings. *dRASSF*^X16/X16^ wings are 8% smaller than control wings. ^∗^p < 0.05 (*white* n = 14, *dRASSF^X16/X16^* n = 11). (F) Histogram representing the number of trichomes in a defined wing area (see Supplemental Experimental Procedures) as a percent of control (*white*). No significant difference was found between control and *dRASSF^X16/X16^* wings (p > 0.05). *white*, n = 430; *dRASSF*, n = 432. Error bars correspond to standard deviations.

Although the *dRASSF* mutant flies are viable, they present a clear growth defect in comparison to wild-type animals when reared in carefully controlled conditions (Figure 1A). *dRASSF* mutant flies were 15% lighter than their wild-type counterparts (Figure 1D), a phenotype which was significantly rescued by introduction of a single copy of a *dRASSF* rescue construct, although wild-type levels of dRASSF were not fully restored (see Figure S1D). *dRASSF* mutant flies were fully fertile and normally proportioned (not shown) but sensitive to γ-irradiation (Figure S1F). Wing surface area was reduced by 8% in *dRASSF* mutant flies, whereas wing hair density was unaffected (Figures 1B, 1C, 1E, and 1F). This suggests that the growth defect of *dRASSF* mutant flies is due to a reduction in cell number and not a defect in cell size.

**Figure 2:**
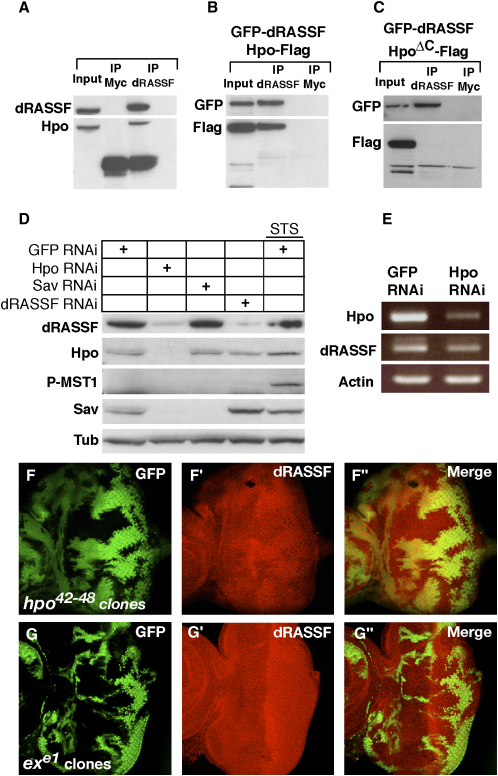
Hpo Interacts with dRASSF and Controls Its Expression Levels (A) Hpo coimmunoprecipitates with dRASSF. Endogenous dRASSF was immunoprecipitated from Kc cells lysates with dRASSF antibodies or control (Myc antibodies). The Membrane was blotted with Hpo66 and dRASSF antibodies. (B) Hpo coimmunoprecipitates with dRASSF in Kc cells. Hpo-Flag and GFP-dRASSF were cotransfected in Kc cells. dRASSF or control (Myc antibodies) immunoprecipitates were blotted for GFP-dRASSF and Hpo-Flag. (C) Hpo lacking its SARAH domain (Hpo^ΔC^) does not interact with dRASSF. Hpo^ΔC^-Flag and GFP-dRASSF were cotransfected in Kc cells. Anti-dRASSF or control (anti-Myc) immunoprecipitates were blotted for GFP-dRASSF and Hpo-Flag. (D) Hpo controls dRASSF and Sav protein levels. Kc cells were treated with GFP, Hippo, Sav, or dRASSF dsRNAs. Lane 5 cells were treated with GFP dsRNA and 3 hr of Staurosporine (STS). Protein extracts were blotted with dRASSF, Hpo66, Sav, P-MST1, and tubulin antibodies. Hpo RNAi strongly reduces both Sav and dRASSF protein levels. dRASSF RNAi stabilizes Sav but is not sufficient to induce Hpo phosphorylation. (E) Hpo loss of function had no effect on dRASSF mRNA expression. RT-PCRs performed on Kc cell lysates treated with GFP or Hpo RNAi. *Hpo*, *dRASSF*, and *Actin* mRNA levels are shown. (F–F″) Hpo controls dRASSF protein levels in vivo. *hpo* mutant clones (marked by a lack of GFP) were generated in eye discs via the *hpo^42–48^* allele. A robust reduction of dRASSF staining (in red [F′]) is observed in *hpo* clones. (G–G″) Ex does not affect dRASSF protein levels. Clones of *ex* mutant cells (marked by a lack of GFP) were generated in eye discs via the *ex^e1^* allele. dRASSF (in red [G′]) staining is unaffected in the clones.

In mammals, members of the RASSF family are known to interact with MST1 and thus to modulate its pro-apoptotic activity [7]. We therefore tested whether dRASSF can interact with Hpo. We performed coimmunoprecipitation (Co-IP) experiments in *Drosophila* Kc cells with dRASSF antibodies to immunoprecipitate endogenous protein. As expected, dRASSF robustly coimmunoprecipitated with Hpo (Figure 2A). The association between Hpo and Sav is mediated by these proteins' shared SARAH domains. Likewise, Hpo's SARAH domain is required for its association with dRASSF, as shown by the fact that a truncated form of Hpo (Hpo^ΔC^) [18] lacking this domain fails to bring down dRASSF (compare Figures 2B and 2C). Thus, the Hpo SARAH domain can associate with both Sav and dRASSF.

Sav is stabilized by the presence of Hpo (18, 19 and Figure 2D, lane 2). We therefore tested whether dRASSF levels are modulated by Hpo. dRASSF immunostaining was reduced in clones mutant for a *hpo* allele that lacks the SARAH domain (Figure 2F). In addition, RNAi-mediated depletion of Hpo from *Drosophila* Kc cells resulted in a reduction of endogenous dRASSF expression (Figure 2D), whereas dRASSF transcripts were unaffected (Figure 2E). By contrast, dRASSF levels were unaffected in clones mutant for other Hpo-pathway members, such as *ex* (Figure 2G), *sav*, and *wts* (Figures S2A and S2B). These results suggest that direct binding to Hpo through its SARAH domain, rather than signaling through the Hpo pathway, is necessary for dRASSF stability. This is analogous to the situation for Sav, which is also stabilized by a kinase-dead form of Hpo [18].

**Figure 3:**
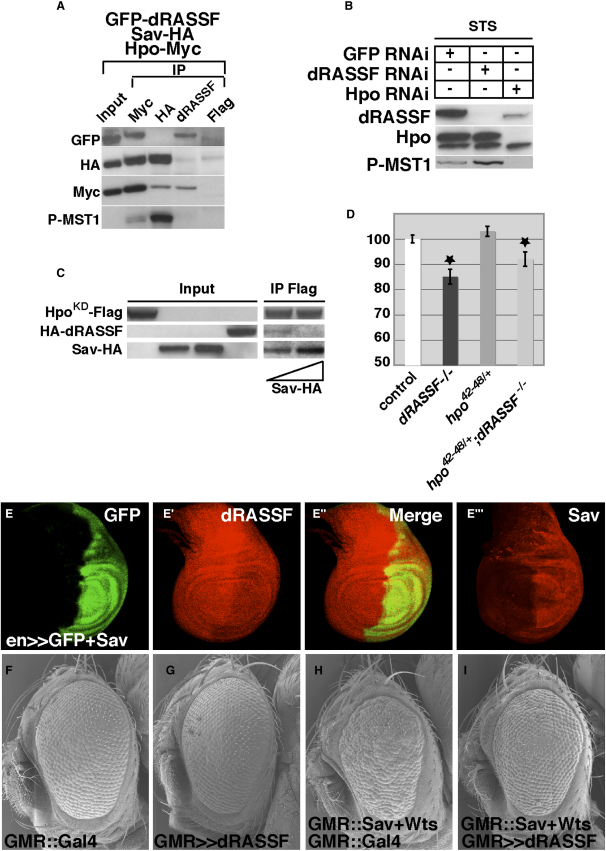
dRASSF Antagonizes Hpo Activity (A) Hpo/Sav and Hpo/dRASSF are two distinct complexes. Hpo-Myc, Sav-HA, and GFP-dRASSF were cotransfected in Kc cells. Anti-Myc, anti-HA, anti-dRASSF, or control (anti-Flag) immunoprecipitates were blotted for GFP-dRASSF, Sav-HA, Hpo-Myc, and phospho-MST1. Hpo interacts with Sav and dRASSF (lane 2). Sav and dRASSF interact only with Hpo and not with each other (lanes 3 and 4). The Hpo fraction bound to Sav is highly phosphorylated (lane 3), and that bound to dRASSF is not (lane 4). (B) dRASSF inhibits Hpo phosphorylation. Kc cells were treated for 4 days with eGFP, dRASSF, or Hpo dsRNAi. The addition of STS 3 hr prior to lysis induced Hpo phosphorylation (lane 1). Western blots were probed with dRASSF, Hpo34, and phospho-MST1 antibodies. In the presence of dRASSF dsRNAi, Hpo phosphorylation increased (lane 2). As expected, the Hpo band disappeared upon Hpo dsRNAi treatment (lane 3). (C) Sav competes with dRASSF to bind Hpo. Kc cell lysates expressing, respectively, Hpo^KD^-Flag, HA-dRASSF, Sav-HA (200 ng), and Sav-HA (400 ng) were mixed, incubated overnight, and immunoprecipitated with Flag antibody. Blots were probed with HA and Flag antibodies. Increasing the amount of Sav displaced dRASSF from Hpo^KD^ (compare lanes 5 and 6). (D) The *dRASSF* phenotype is sensitive to *hpo* loss of function. The histogram represents the total body weight as a percent of control flies (*white*). The reduction in body size in *dRASSF* flies can be partially rescued by removal of one copy of *hpo* (*hpo^42–48^* allele). ^∗^p < 0.05 (*white* n = 80, *dRASSF^X16/X16^* n = 80, *FRT42D, hpo^42–48^*/+ n = 80, and *FRT42D, hpo^42–48^*/+; *dRASSF^X16/X36^* n = 80). (E–E″′) Sav controls dRASSF protein level. GFP (in green) and Sav were expressed in the posterior half of the wing disc by the *engrailed-GAL4 (en-GAL4*) driver. A robust reduction of dRASSF staining (in red [E′]) was observed in the *en* domain. [E″′] shows Sav overexpression in a separate disc. (F–I) *dRASSF* reduces apoptosis induced by *sav* and *wts* coexpression. Shown are scanning electron micrographs of *Drosophila* heads from *GMR::Gal4* control animals (F) or from *GMR::Gal4/UAS::dRASSF* (G), *GMR::Gal4;GMR::sav+wts* (H), or (I) *GMR::Gal4/UAS::dRASSF*;*GMR::sav+wts* (I). Overexpression of dRASSF inhibits the rough-eye phenotype generated by coexpression of Sav and Wts. See Supplemental Experimental Procedures for exact genotypes. Error bars correspond to standard deviations.

Because Hpo, Sav, and dRASSF all contain a SARAH domain, we speculated that dRASSF might also bind Sav. To test this, we investigated whether dRASSF interacts with Sav by co-IP but repeatedly failed to detect such an interaction (Figure S2C and data not shown). Because the possibility of a ternary complex had been raised by Scheel and Hofmann [13], we then tested whether the three proteins could be found in the same complex. We coexpressed Hpo, Sav, and dRASSF in cultured Kc cells. As expected, Hpo was able to bind Sav and dRASSF (Figure 3A). However, Sav immunoprecipitates only contained Hpo and not dRASSF, and dRASSF immunoprecipitates contained Hpo but not Sav (Figure 3A). We obtained identical results with endogenous IPs by using dRASSF and Sav antibodies (Figure S2C). These data support the notion that Sav and dRASSF are not present in the same complex but are in two different Hpo complexes.

Sav has been shown to be a positive regulator of the Hpo pathway, whereas our genetic results suggest that dRASSF might antagonize Hpo function. We were therefore interested in determining whether complexing with Sav or dRASSF might influence Hpo activity. We probed our immunoprecipitates with an phospho-MST1 antibody that recognizes phosphorylated (active) Hpo [20]. Interestingly, although Hpo that was coimmunoprecipitated with dRASSF showed barely detectable levels of phosphorylation, the Sav-associated fraction was highly phosphorylated (Figure 3A). Thus, Hpo can exist as two pools, a highly active Sav-associated pool and an inactive dRASSF-associated pool. This correlates with data showing that Nore1 can repress MST1 activity in mammalian cells [16]. This also suggests that Sav can promote Hpo activation and provides the first direct evidence of a function for the Hpo/Sav interaction.

Next, we wanted to test our prediction that dRASSF depletion would promote Hpo activation. Like that of Hpo's mammalian counterparts, phosphorylation of endogenous Hpo can be potently stimulated by the drug Staurosporine (STS) in Kc cells (16, 20, 21 and Figure 3B, lane 1). Although RNAi depletion of dRASSF alone was not able to induce Hpo phosphorylation (Figure 2D, compare lanes 4 and 5), dRASSF depletion markedly potentiated STS-induced Hpo activation (Figure 3B, compare lanes 1 and 2). Thus, dRASSF restricts Hpo activation in cultured cells.

Given their opposing effects on Hpo activation, we investigated the relationship between Sav and dRASSF. Depletion of dRASSF in Kc cells gives rise to an increase in Sav protein levels (Figure 2D lines 1 and 4). Although dRASSF levels were unaltered in *sav* mutant clones (Figure S2A), overexpression of Sav in the wing disc results in a robust decrease of dRASSF staining (Figure 3E). We then tested whether dRASSF and Sav compete to bind Hpo. To address this question, because Sav and dRASSF repress each other's expression and dRASSF has reduced affinity for phosphorylated Hpo, we mixed separate Kc cell lysates expressing a kinase-dead form of Hpo (Hpo^KD^-Flag), Sav-HA, and HA-dRASSF and performed IPs after the proteins were allowed to bind overnight. Both Sav and dRASSF were able to interact with Hpo (Figure 3C). In these conditions, increasing the amount of Sav was able to displace the dRASSF fraction bound to Hpo, showing that Sav and dRASSF are competing to bind Hpo. The outcome of the competition probably determines the stability of Sav and dRASSF; both proteins are downregulated when Hpo is depleted by RNAi (Figure 2D). Thus, we suggest that interplay between the inhibitor dRASSF and the activator Sav determines the level of Hpo activation and therefore affects body size.

We tested this model by performing genetic-interaction experiments. We crossed a mutant allele of *hpo* into the *dRASSF* mutant background and measured the adult body mass (Figure 3D). The body-mass reduction of dRASSF mutant flies (15% reduction) was substantially rescued by removal of just one copy of Hpo (8% reduction). Flies overexpressing Sav showed a reduction of 10% in weight and 5% in wing area, mimicking dRASSF loss of function (Figure S3). This wing defect was significantly increased in a dRASSF mutant background (Figures S3B–S3D). In addition, misexpression of dRASSF was able to robustly rescue the rough-eye phenotype elicited by coexpression of Sav and Wts (Figures 3F–3I). These data support the notion that dRASSF can antagonize Sav-mediated Hpo activation in vivo.

**Figure 4:**
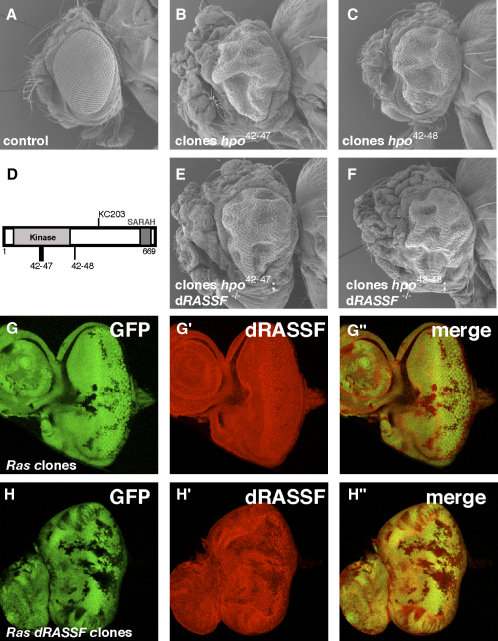
Tumour-Suppressor Function of *dRASSF* (A–C, E, and F) Scanning Electron Micrographs of *Drosophila* heads from control animals (A), animals bearing *hpo^42–47^* clones (B), *hpo^42–48^* clones (C), *hpo^42–47^* clones in a dRASSF loss-of-function background (E), or *hpo^42–48^* clones in a dRASSF loss-of-function background (F). The overgrowth phenotype elicited by the loss of *hpo* is enhanced by the removal of *dRASSF*. See Supplemental Experimental Procedures for genotypes. (D) Schematic representation of Hpo protein showing the different mutations used. The *hpo^42–47^* allele causes a deletion of six amino acids in the kinase domain, and this deletion probably inhibits Hpo-ATP binding. The *hpo^42–48^* allele is a deletion of 20 bp and gives rise to a premature stop codon. *hpo^KC203^* changes G to A at the 5′ splicing site and the translation run into a stop codon in the intron. (G–H″) *dRASSF* rescues *Ras1* loss of function. (G–G″) *Ras1^c40b^* clones (marked by a lack of GFP) are small. (H–H″) *Ras^c40b^ dRASSF^X36^* clones (marked by a lack of GFP) are larger than *Ras^c40b^* clones. dRASSF staining is in red (G′ and H′).

Though our results are consistent with biochemical data on mammalian RASSF family members 7, 16, they are at odds with the fact that *RASSF* genes are commonly silenced in tumor cells. Avruch and colleagues have proposed that one RASSF protein, Nore1, possesses a tumor-suppressor function that is independent of MST1 and MST2 [22]. We found two lines of evidence to support this notion. First, we made clones that are mutant for two *hpo* hypomorphic alleles, *hpo^42–48^* ([19] compare Figures 4C and 4F), *hpo^KC203^* ([23] not shown), that remove the SARAH domain in a *dRASSF* mutant background in the head by using the eyeless FLP system [24]. Interestingly, the overgrowth phenotype elicited by these *hpo* alleles was strongly enhanced by loss of *dRASSF*. By contrast, a *hpo* allele (*hpo^42–47^* [19]) bearing an inactivating deletion in the kinase domain but an intact SARAH domain was barely if at all enhanced by *dRASSF* loss of function (Figures 4B and 4E). This suggests that dRASSF may possess a tumor-suppressor function, which may be uncovered when the Hpo function is compromised.

In addition, we examined the relationship between Ras1 and dRASSF because the mammalian RASSF proteins have all been shown to bind Ras proteins 8, 9, 10, 25. In *Drosophila* imaginal tissues, *Ras1* mutant clones grow poorly and are eliminated by apoptosis (26, 27 and Figure 4G). When we made double-mutant clones for *Ras1* and *dRASSF* in the developing eye, we observed a substantial rescue of the growth defect observed in clones mutant for *Ras1* alone (Figure 4H). This rescue of *Ras* loss of function was the result of both increased proliferation (Figure S4) quantified with phosphorylated Histone 3 staining and a reduction of apoptosis visualized with a cleaved-Caspase 3 antibody (Figure S5). Thus, *dRASSF* appears to antagonize Ras1 signaling in growth control, which is again suggestive of a “tumour-suppressing” effect distinct from its “oncogenic” role in opposing the Hpo pathway. However, Aoyama et al. suggest that NORE1 may also have both Ras- and MST-independent functions [22]. Future experiments will therefore be aimed at gaining a better understanding of the RASSFs' growth-restricting functions. The fact that the *dRASSF* mutations are viable might therefore reflect the facts that its ability to regulate the Hpo pathway may be redundant with other modes of regulation and that loss of dRASSF's tumor-suppressive activity is balanced by loss of its growth-promoting activity. O'Neill et al. have proposed that MST2 may be inactivated by binding to Raf-1. It will be interesting to determine whether this mode of regulation is redundant with RASSF [28].

In summary, we have generated mutant alleles of the sole *Drosophila* ortholog of the *RASSF* family of tumor suppressors. Surprisingly, *dRASSF* mutant flies are smaller than control flies. This growth defect can probably be ascribed in part to dRASSF's ability to antagonize Hpo signaling by competing with Sav for binding to Hpo. In addition, we have shown that dRASSF also possesses a tumor-suppressor activity, which is uncovered when *hpo* or *Ras1* function is compromised. It will be interesting to investigate whether some mammalian RASSF proteins share these properties.

## Supplemental Data

Document S1. Experimental Procedures and Five Figures
