# Supplementary material for: The Drosophila RASSF Homolog Antagonizes the Hippo Pathway
Source: Curr Biol. 2006 Dec 19;16(24):2459–65. doi: 10.1016/j.cub.2006.10.060 (PMC1828611; doi:10.1016/j.cub.2006.10.060)
Supplement: Document S1. Experimental Procedures and Five Figures [file mmc1.pdf]

Supplemental Data

The *Drosophila* RASSF Homolog Antagonizes the Hippo Pathway

Cedric Polesello, Sven Huelsmann,
Nicholas H. Brown, and Nicolas Tapon

Supplemental Experimental Procedures

Generation and Characterization of dRASSF Alleles

GE23517 (from Genexel) and EY2800 P elements are viable without apparent phenotypes. Excisions of GE23517 were screened by PCR over the original P element with the following primers: 5′-CGATCGATTGTTTACGTTCCGCTGTGC-3′ and 5′-GGGCAGCAGGAGATATGGTGATTAGTCG-3′. Sequencing of the PCR products revealed deletions of 932 bp and 620 bp starting at the P element insertion for the *dRASSF*^{X16} and *dRASSF*^{X36} alleles, respectively. *dRASSF*^{X51} is a 1924 bp deletion removing sequences of both sides of the original P elements. *dRASSF*$^{44.2}$ was generated by mobilization of EY2800. To map the deletion of *dRASSF*$^{44.2}$, we obtained genomic DNA from homozygous flies and amplified the *dRASSF* locus by using the primers 5′-CATCAACGCCCGGGCTTGCAGTGTA-3′ and 5′-GTCAGCACGGTTTATCAGTGTTGG-3′. Sequencing of the PCR product revealed a deletion of 2120 bp including the start sites of transcription and translation. We furthermore confirmed by reverse transcriptase polymerase chain reaction (RT-PCR) that the expression of the neighboring *cenB-1A* gene was not visibly affected.

Genomic Rescue Construct pWRpA-dRASSF and pAW-dRASSF

A 7 kb Spe1-BamH1 fragment of the BAC 17P04 containing the CG4656 locus was cloned into pBluescript, verified, and subsequently cloned into the pWhiteRabbitpolyA transformation vector (N.H.B., unpublished data).

We cloned dRASSF into the Gateway pActin5C vectors (3× HA and GFP from *Drosophila* Gateway Vector Collection) by using PCR fragments from EST LD40758 (from DGRC) and the following primers: 5′-CAACATGTGGAAGTGCCACAAGTGCG-3′ and 5′-GTTTTACAAATGCACTTTCAGAGATTCC-3′.

RT-PCR

mRNA extraction from cells and larvae extracts was performed with the RNeasy Mini Kit (Qiagen). RT was performed with 2 μg of total mRNA with the Invitrogen Superscript III kit. Two microliters of the templates was used for performing the PCR reaction. Primers used for PCR were as follows: RNAif Hpo: 5′-CTAATACGACTCACTATAGGGAGTCCGCAGAAGCCACCATCG-3′; RNAir Hpo, 5′-CTAATACGACTCACTATAGGGAGCCAAGTTCGACTCCAGCTCCACC-3′; dRASSFf, 5′-CTCAAATGTCAGTGTGGGTCTCCTCG-3′; dRASSFr, 5′-GCCTGGAATTGATTGTTAGTTTCGG-3′; ActinR, 5′-CACCCTGAAGTACCCCATTGAGCA-3′ C-30; and ActinF, 5′-CAGACGCAGGATGGC ATGGGGAAG G-3′.

Fly Stocks

yw;FRT42D,hpo$^{42–47}$*/Cyo*, *yw;FRT42D,hpo*$^{42–48}$*/Cyo*, *yw;FRT42D, hpo*^{KC203}*/Cyo*, *yw eyFLP;+;FRT82B,sav*^{3}*/TM6b*, and *yw;FRT82B, wts*^{latsX1}*/TM6b* were previously described [S1–S4]. *yw;FRT42A, ex*^{e1}*/Cyo* was a kind gift from Iswar K Hariharan [S5]. *yw hsFLP;UAS Hpo* was previously described [S6]. Other stocks were from the Bloomington *Drosophila* Stock Center.

Genotypes were as follows:

Figure 1A: *w* and *w; dRASSF*^{X16}
Figure 1B: *w*
Figure 1B′: *w; dRASSF*^{X16}
Figure 1C: *w and w; dRASSF*^{X16}
Figure 1D: *w, w; dRASSF*^{X16}, *w; dRASSF*$^{X16/X36}$, *w; dRASSF*$^{X16/44.2}$, *w; pWRpA-dRASSF; dRASSF*$^{X16/44.2}$
Figures 1E and 1F: *w and w; dRASSF*^{X16}
Figure 2F: *yw eyFLP; FRT42D, ubiGFP/FRT42D, hpo*$^{42–48}$
Figure 2G: *yw eyFLP; FRT40A, ubiGFP/ FRT40A, ex*^{e1}
Figure 3E: *w; en::Gal4, UAS::GFP/ UAS::Sav*
Figure 3F: *yw/w; GMR::Gal4/+*
Figure 3G: *yw/w; GMR::Gal4/+; UAS::dRASSF*
Figure 3H: *yw/w; GMR::Gal4/+; GMR::Sav,GMR::Wts/+*
Figure 3I: *yw/w; GMR::Gal4/+; GMR::Sav,GMR::Wts/UAS::dRASSF*
Figure 4A: *w*
Figure 4B: *yw eyFLP; FRT42D, ubiGFP/FRT42D, hpo*$^{42–47}$
Figure 4C: *yw eyFLP; FRT42D, ubiGFP/FRT42D, hpo*$^{42–48}$
Figure 4E: *yw eyFLP; FRT42D, ubiGFP/FRT42D, hpo*$^{42–47}$*; dRassf*$^{X36/X36}$
Figure 4F: *yw eyFLP; FRT42D, ubiGFP/FRT42D, hpo*$^{42–48}$*; dRassf*$^{X36/X36}$
Figure 4G: *yw eyFLP; FRT82B, ubiGFP/FRT82B, dRas*^{c40b}
Figure 4H: *yw eyFLP; FRT82B, ubiGFP/FRT82B, dRas*^{c40b}*, dRASSF*^{X36}
Figure S1E: *yw eyFLP; FRT82B, ubiGFP/FRT82B, dRASSF*^{X36}
Figure S2A: *yw eyFLP; FRT82B, ubiGFP/FRT82B, sav*^{3}
Figure S2B: *yw eyFLP; FRT82B, ubiGFP/FRT82B, wts*^{latsX1}
Figure S3A: *w, MS10696::Gal4/+*
Figure S3B: *w, MS10696::Gal4/+; UAS::Sav/+*
Figure S3C: *w, MS10696::Gal4/+; UAS::Sav/+; dRASSF*$^{X16/44.2}$
Figure S4A: *yw eyFLP; FRT82B, ubiGFP/FRT82B*
Figure S4B: *yw eyFLP; FRT82B, ubiGFP/FRT82B, dRASSF*^{X36}
Figure S4C: *yw eyFLP; FRT82B, ubiGFP/FRT82B, dRas*^{c40b}
Figure S4D: *yw eyFLP; FRT82B, ubiGFP/FRT82B, dRas*^{c40b}*, dRASSF*^{X36}
Figure S5A: *yw eyFLP; FRT82B, ubiGFP/FRT82B*
Figure S5B: *yw eyFLP; FRT82B, ubiGFP/FRT82B, dRASSF*^{X36}
Figure S5C: *yw eyFLP; FRT82B, ubiGFP/FRT82B, dRas*^{c40b}
Figure S5D: *yw eyFLP; FRT82B, ubiGFP/FRT82B, dRas*^{c40b}*, dRASSF*^{X36}

Standard Growth Conditions and Larval Irradiation

For each experiment, 50 L1 larvae (0–2 hr collections) were collected 24 hr after egg deposition (AED). Weight measurements of adult flies were performed as described [S7]. Relative wing and cell sizes were quantified, and cell number values were calculated as described [S8]. Images of adult flies were obtained with a Nikon DXM1200 digital camera on a Leica MZFL III microscope. Wing pictures were obtained with a Zeiss Axioplan microscope. Averages of triplicate and duplicate experiments are presented, respectively, in Figures 1 and 3.

L3 larvae were irradiated with 40 Gy of γ rays and reared in standard growth conditions.

Kc Cell Assays

In depletion experiments *Drosophila* embryonic Kc cells were treated with dRASSF, Hpo, or eGFP dsRNAs for 4 days. dsRNAs were synthesized with T7 Ribomax large-scale RNA production systems (Promega) and purified with the MEGAclear system (Ambion). Treating Kc cells with 1 μM staurosporine (Sigma) for 3 hr induced Hpo phosphorylation. Transfections were done with Effectene (Qiagen).

RNAi Primers

RNAif dRASSF: 5′-CAATGGCCCGCCACTCCCACGTGATCAC-3′
RNAir dRASSF: 5′-CCGTCGTGGTCGTATCCTCGTCGTCGTTGC-3′
RNAif Hpo: 5′-CTAATACGACTCACTATAGGGAGTCCGCAGAAGCCACCATCG-3′
RNAir Hpo: 5′-CTAATACGACTCACTATAGGGAGCCAAGTTCGACTCCAGCTCCACC-3′
RNAIf Sav: 5′-GCACACACAACTGGCAGCTCACC-3′
RNAir Sav: 5′-CGTGGGTGGGGCATAGAGCGAC-3′
RNAif eGFP: 5′-CTAATACGACTCACTATAGGGAGTGGTGGTGCCCATCCTGGT-3′
RNAir eGFP: 5′-CTATACGACTCACTATAGGGAGTTCGCGCTTCTCGTTGGGG-3′

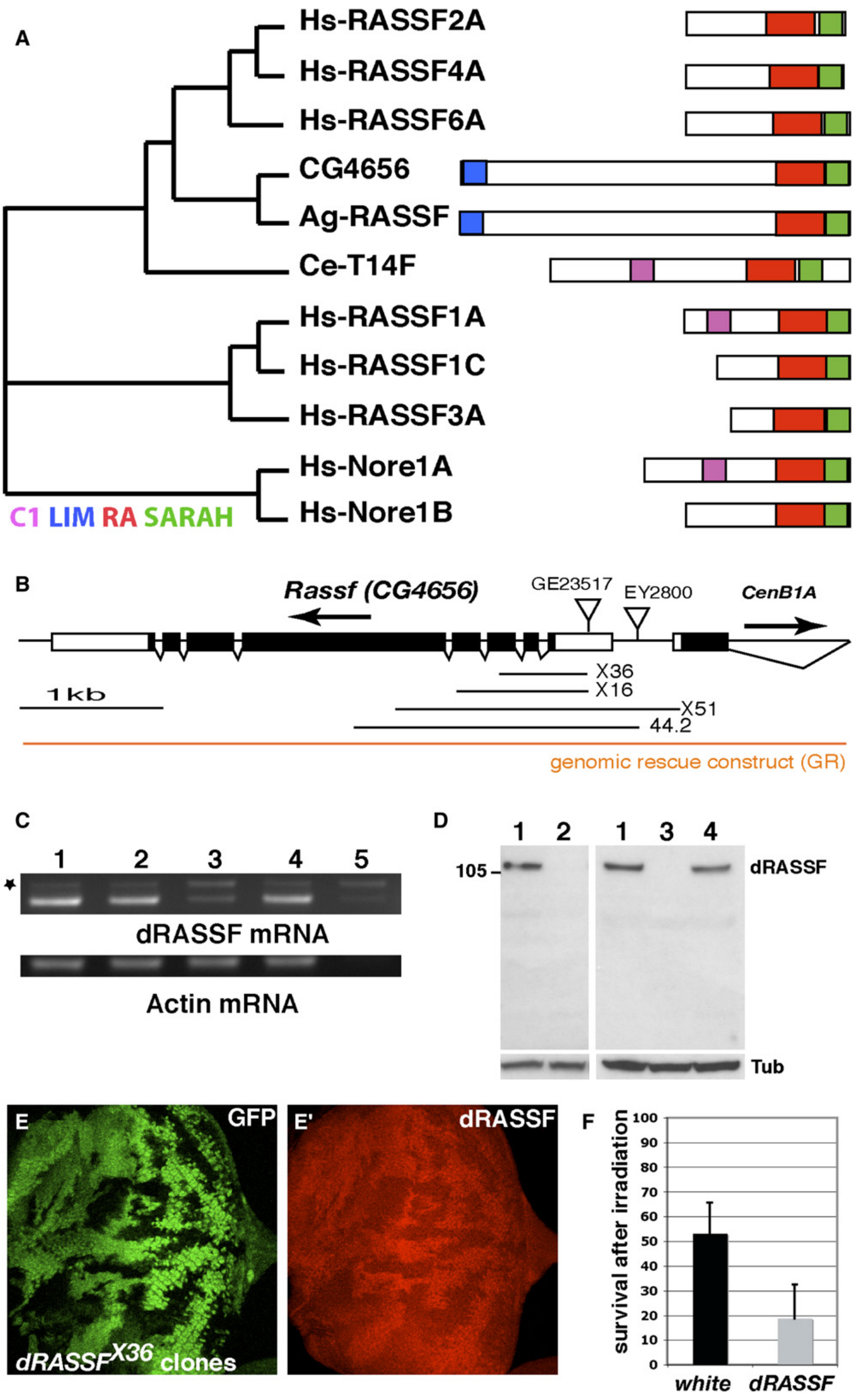

Figure S1. dRASSF Protein Family Phylogeny and the *dRASSF* Locus

(A) Phylogenetic tree of the RASSF protein family. The RASSF protein family is composed of six members in humans. The protein numbers are the following: NP_009113 for RASSF1A, NP_733831 (RASSF1C), NP_872604 (NORE1A), NP_872605 (NORE1B), NP_739580 (RASSF2A), NP_835463 (RASSF3A), AAO61138 (RASSF4A), and AAO61689 (RASSF6A). Only one RASSF protein is found in *Drosophila* (translation of LD40758), *C. elegans* (NP_001022361), and *Anopheles* (EAA6455). Protein alignments were performed with Clustal W, and tree generation was performed with Tree View86K. (B) *Drosophila RASSF* genomic locus. *dRASSF*^{X16}, *dRASSF*^{X36} and *dRASSF*^{X51} were generated by mobilization of the GE23517 P element, and *dRASSF*$^{44.2}$ was generated by EY2800 mobilization. The sizes of the deletions are indicated as black lines. The genomic rescue construct is shown in orange.

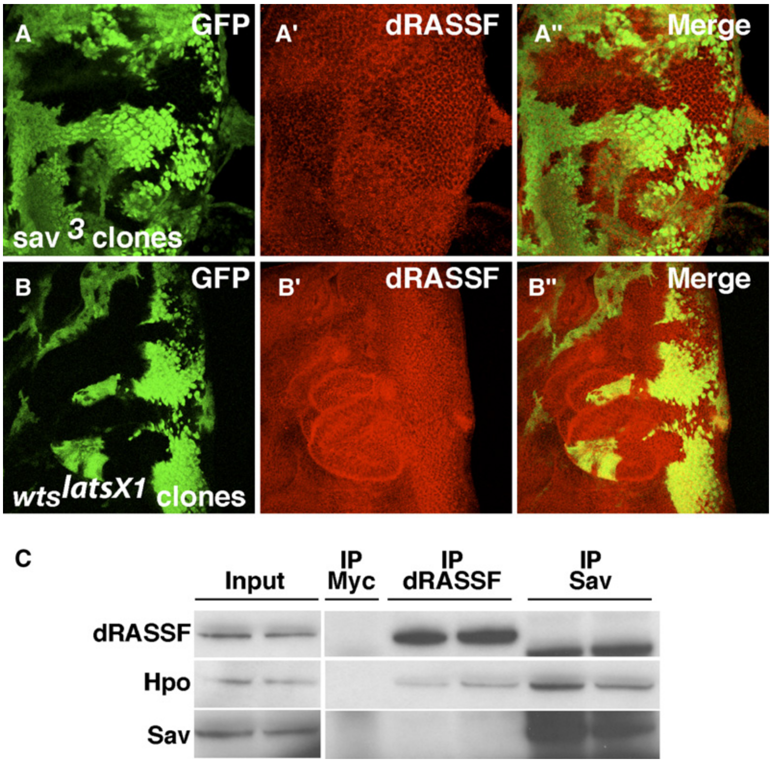

Figure S2. *sav* and *wts* Mutations Do Not Alter dRASSF Levels

(A and B) Third-larval-instar eye-imaginal discs stained with anti-dRASSF (red). In sav^{3} (A) and wts^{latsX1} (B) mutant cells (lack of GFP), dRASSF protein levels are not changed.

(C) Sav/Hpo and dRASSF/Hpo are two distinct complexes. Kc cell lysates were immunoprecipitated with Myc, dRASSF, or Sav antibodies and blotted with dRASSF, Hpo66, and Sav antibodies. Whereas Hpo binds dRASSF and Sav, Sav did not bring down dRASSF, nor did dRASSF bring down Sav. Duplicate IPs are shown.

Western Blotting and Immunoprecipitation

For western blotting, twenty eye-brain complexes of control and mutant flies were dissected in Schneider's medium + 10% FCS. Tissues or Kc cells were lysed on ice in 50 and 200 μl, respectively, of ice-cold lysis buffer (150 mM NaCl, 50 mM Tris [ph 8], 0.5% NP40, 1 mM EGTA, 0.5 M NaF, 200 mM vanadate, phosphatase inhibitor cocktail 1 from Sigma, and protease inhibitors from Roche). After material was cleared by centrifugation at 14000 rpm for 10 min, samples containing 50 μg of protein were resolved by SDS-PAGE (NuPAGE 4%–12% Bis-Tris gels from Invitrogen), and proteins were transferred to PVDF membranes and visualized by immunoblotting with phospho-Mst1 (Thr183)/Mst2 (Thr180) (1/1000 from Cell Signaling Technology), Hpo34 (1/1000 dilution [S9]), Hpo66 (1/1000), dRASSF59 (1/10000), dRASSF60 (1/5000), and Sav (1/2500) antibodies. The Rabbit anti-dRASSF59 and -dRASSF60 antibodies were raised against peptides corresponding to amino acids 792–806 and 294–308 of dRASSF, respectively. Anti-rabbit Sav was raised against amino acids 9–24. Rat anti-Hpo66 was raised against amino acids 248–262. Anti-Myc (mouse c-*myc* 9E10): sc-40 and rabbit c-*myc* (A-14): sc-789 from Santa Cruz Biotechnology, 1/5000), anti-HA (clone 3F10 from Roche, 1/5000), anti-Flag (F1804 from Sigma), and anti-GFP generated by the Cancer Research UK monoclonal antibody service were used. Anti-tubulin was used for normalization (Developmental Biology Hybridoma Bank, 1/5000). Chemoluminescence was observed via an ECL plus western-blotting detection system (Amersham Biosciences). For all western-blotting experiments, the same membrane was probed first with phospho-Hpo antibody and then with Hpo, dRASSF, tags, and tubulin antibodies after stripping.

Immunoprecipitations were done on 250 μg of protein from transfected lysates and 500 μg of protein for endogenous IPs in lysis buffer for 2 hr at 4°C with rabbit anti-c-*myc* (A-14): sc-789 from Santa Cruz Biotechnology,1/5000), rabbit anti-dRASSFs (1/600), rabbit anti-Sav (1/300), rabbit anti-Flag (F7425 Sigma, 1/300), and rabbit anti-HA.11 (MMS-101R, Covance, 1/300) as previously described [S6].

Genetics and Immunocytochemistry

Mosaic tissues were obtained with the FLP/FRT system with eyFLP drivers. Tissues were dissected in 1× PBS, fixed in 4% formaldehyde in PBS for 20 min at room temperature, washed in PBS containing 0.1% Triton X-100 (PBT), transferred in PBT 0.3% Triton for 30 min, and washed again in PBT. Tissues were then blocked for two hours in PBT containing 10% goat serum. Primary antibodies were incubated overnight at 4°C. dRASSF antibodies (59 and 60) were used at 1/200, Sav antibody at 1/100, and phospho-Histone H3 (Upstate) and activated Caspase3 (Cell Signaling) antibodies at 1/500. Secondary antibodies (Rhodamine Red X donkey anti rabbit from Jackson ImmunoResearch) were incubated for 2 hr at room temperature at 1/500. After washes, tissues were mounted in Vectashield (Vector). Fluorescence images were acquired on a Zeiss LSM510 Meta confocal laser scanning microscope (25× and 40× objective lenses).

(C) RT-PCRs were performed on *Drosophila* larval extracts. *dRASSF* and *Actin* mRNA levels are shown. (lane 1) *white*, (lane 2) $dRASSF^{X36}$, (lane 3) $dRASSF^{44.2}$, (lane 4) $dRASSF^{X16/44.2}$, (lane 5) negative control, *white* minus reverse transcription. A robust reduction of dRASSF mRNA was observed in $dRASSF^{44.2}$ alleles but not in $dRASSF^{X16}$ and $dRASSF^{X36}$. *Genomic band product.

(D) Western blot performed on larval eye-brain complexes. The anti-dRASSF antibodies recognize endogenous dRASSF protein in control larvae at the expected molecular weight. This band disappears in *dRASSF* mutant larvae. (1) *white*, (2) $dRASSF^{44.2}$, blotted with the dRASSF59 antibody. Tubulin staining is shown as a loading control. (3) $dRASSF^{X16/44.2}$, (4) genomic rescue construct expression in a $dRASSF^{X16/44.2}$ mutant background; western blotting was performed with dRASSF60 and tubulin antibodies.

(E and E′) Immunostaining with dRASSF antibody in eye discs containing $dRASSF^{X36}$ mutant cells (GFP-negative cells).

(F) Histogram representing survival rates (in percent) to adulthood after γ-irradiation (40 Gy). *dRASSF* flies are less resistant to irradiation than controls. *white* n = 150, $dRASSF^{X16}$ n = 400. n = number of L3 larvae irradiated.

Error bars correspond to standard deviations.

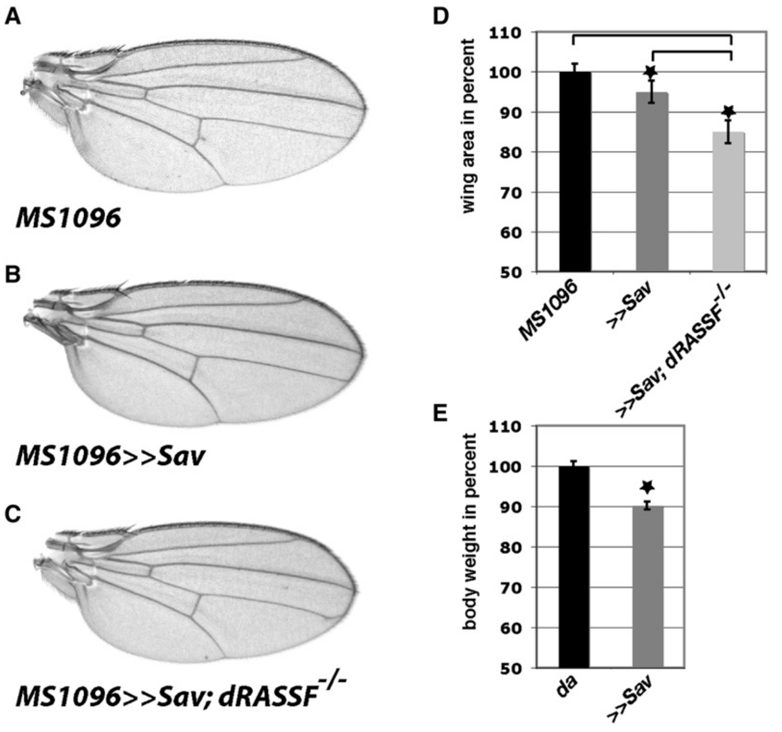

Figure S3. *dRASSF* Mutations Enhance the Phenotypes Elicited by *sav* Misexpression

(A–C) Adult wings. (A) *MS1096::Gal4* wing-specific driver. (B) *MS1096::Gal4; UAS::Sav.* (C) *MS1096::Gal4; UAS::Sav;dRASSF*$^{X16/44.2}$.

(D) Histogram representing the wing area as a percentage of control (*MS1096* flies). Overexpression of *sav* gives rise to a 5% reduction in wing area; this is compared with a 15% reduction in a *dRASSF* mutant background. *MS1096* n = 9, *MS1096>>sav* n = 11, and *MS1096>>sav; dRASSF*$^{-/-}$ n = 9. See Supplemental Experimental Procedures for exact genotypes. $*p < 0.05$.

(E) Histogram showing adult-fly body masses as a percentage of control. Overexpression of *sav* with the ubiquitous *da::Gal4* driver reduces the weight by 10%. *da::Gal4* n = 114, *da>>sav* n = 108. $*p < 0.05$.

Error bars correspond to standard deviations.

Clone Size and Mitotic-Index Quantification

GFP-positive and -negative areas were separated with Image J; the same macro as that described in Colombani et al. [S9] was used. Clones areas were measured with Image J histogram function and Histone H3-positive cells were quantified with the Image J cell counter.

SEM Procedures

Scanning microscopy (SEM) of adult flies was performed as described in [S10].

Supplemental References

S1. Wu, S., Huang, J., Dong, J., and Pan, D. (2003). *hippo* encodes a Ste-20 family protein kinase that restricts cell proliferation and promotes apoptosis in conjunction with salvador and warts. Cell *114*, 445–456.

S2. Udan, R.S., Kango-Singh, M., Nolo, R., Tao, C., and Halder, G. (2003). Hippo promotes proliferation arrest and apoptosis in the Salvador/Warts pathway. Nat. Cell Biol. *5*, 914–920.

S3. Xu, T., Wang, W., Zhang, S., Stewart, R.A., and Yu, W. (1995). Identifying tumor suppressors in genetic mosaics: the Drosophila lats gene encodes a putative protein kinase. Development *121*, 1053–1063.

S4. Tapon, N., Harvey, K.F., Bell, D.W., Wahrer, D.C., Schiripo, T.A., Haber, D.A., and Hariharan, I.K. (2002). *salvador* Promotes both cell cycle exit and apoptosis in *Drosophila* and is mutated in human cancer cell lines. Cell *110*, 467–478.

S5. Boedigheimer, M., and Laughon, A. (1993). Expanded: A gene involved in the control of cell proliferation in imaginal discs. Development *118*, 1291–1301.

S6. Pantalacci, S., Tapon, N., and Leopold, P. (2003). The Salvador partner Hippo promotes apoptosis and cell-cycle exit in Drosophila. Nat. Cell Biol. *5*, 921–927.

S7. Meyer, C.A., Jacobs, H.W., Datar, S.A., Du, W., Edgar, B.A., and Lehner, C.F. (2000). Drosophila Cdk4 is required for normal growth and is dispensable for cell cycle progression. EMBO J. *19*, 4533–4542.

S8. Rulifson, E.J., Kim, S.K., and Nusse, R. (2002). Ablation of insulin-producing neurons in flies: Growth and diabetic phenotypes. Science *296*, 1118–1120.

S9. Colombani, J., Polesello, C., Josue, F., and Tapon, N. (2006). Dmp53 activates the Hippo pathway to promote cell death in response to DNA damage. Curr. Biol. *16*, 1453–1458.

S10. Kango-Singh, M., Nolo, R., Tao, C., Verstreken, P., Hiesinger, P.R., Bellen, H.J., and Halder, G. (2002). Shar-pei mediates cell proliferation arrest during imaginal disc growth in Drosophila. Development *129*, 5719–5730.

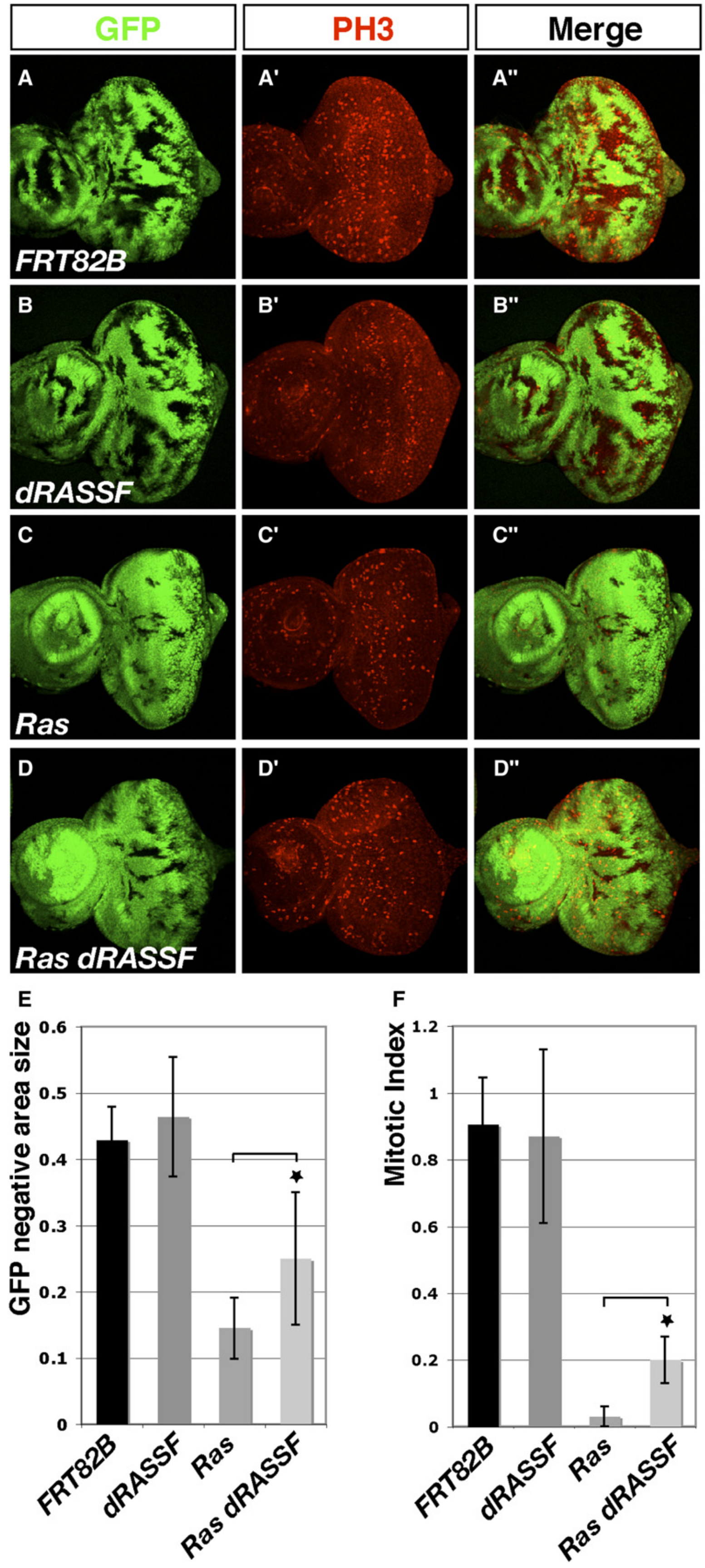

Figure S4. *dRASSF* **Loss of Function Partially Restores the Proliferation rate of** *Ras* **Mutant Cells**

(A–D″) Eye imaginal discs stained with anti-phosphorylated Histone H3. (A) *FRT82B* clones. (B) *FRT82B* $dRASSF^{X36}$ clones. (C) *FRT82B* Ras^{c40b} clones. (D) *FRT82B* Ras^{c40b}, $dRASSF^{X36}$ clones.

(E) Histogram representing the size of the GFP-negative areas versus total eye-disc area. No significant difference was observed between *FRT82B* clones and *dRASSF* clones. *dRASSF* mutation partially rescues the *Ras* clonal growth defect. *FRT82B* n = 18, *dRASSF* clones n = 16, *Ras* clones n = 12, and *Ras dRASSF* clones n = 12. n = number of discs $^*p < 0.05$

(F) Histogram showing the mitotic index in the different genotypes shown in (A)–(D). *dRASSF* mutant cells behave like control cells. The *dRASSF* mutation significantly rescues the proliferation rate of *Ras* mutant cells. *FRT82B* n = 10, *dRASSF* clones n =6, *Ras* clones n = 6, and Ras *dRASSF* clones n = 6. n = number of discs. $^*p < 0.05$.

Error bars correspond to standard deviations.

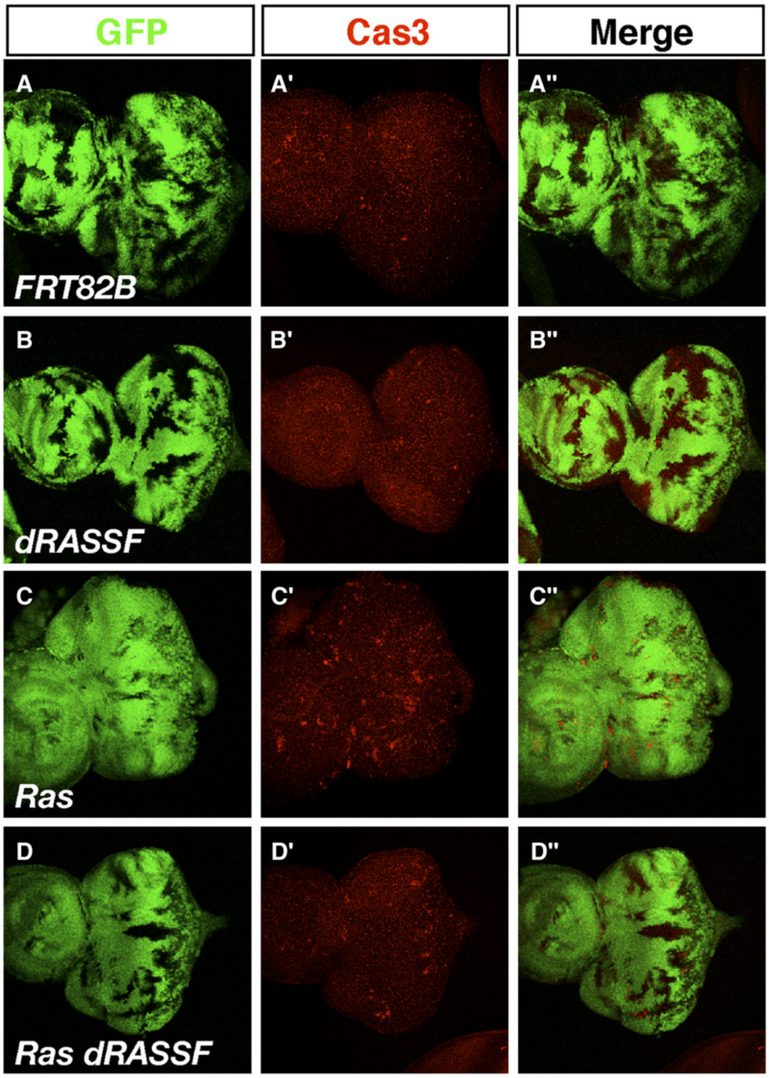

Figure S5. ***dRASSF*** **Loss of Function Partially Rescues Cell Death of** ***Ras*** **Mutant Tissue**

(A–D) Larval eye discs stained with a cleaved-Caspase 3 antibody. (A) *FRT82B* clones. (B) *FRT82B dRASSF*^{X36} clones. (C) *FRT82B Ras*^{c40b} and *FRT82B Ras*^{c40b}, *dRASSF*^{X36} clones.
